# An Integrated Multidisciplinary Circuit Led by Hospital and Community Pharmacists to Implement Clopidogrel Pharmacogenetics in Clinical Practice

**DOI:** 10.3390/pharmacy11020076

**Published:** 2023-04-17

**Authors:** Joan Francesc Mir, Cristina Rodríguez-Caba, Maria Estrada-Campmany, Edurne Fernández de Gamarra-Martínez, Maria Antònia Mangues, Guillermo Bagaría, Pau Riera

**Affiliations:** 1 Professional Projects and Research Area, Col·legi de Farmacèutics de Barcelona/Barcelona Pharmacists’ Association, 08009 Barcelona, Spain; mir.bonnin@cofb.net (J.F.M.); rodriguez.caba@cofb.net (C.R.-C.); mestrada@cofb.cat (M.E.-C.); gbagaria001@cofb.net (G.B.); 2 Blanquerna School of Health Sciences, Universitat Ramon Llull, 08022 Barcelona, Spain; 3 Pharmacy Department, Institut d’Investigació Biomèdica Sant Pau (IIB-Sant Pau), Hospital de la Santa Creu i Sant Pau, 08025 Barcelona, Spain; efernandezg@santpau.cat (E.F.d.G.-M.); mmangues@santpau.cat (M.A.M.); 4 CIBER de Enfermedades Raras (CIBERER), Instituto de Salud Carlos III, 28029 Madrid, Spain

**Keywords:** pharmacogenetics, clopidogrel, pharmaceutical care, clinical pharmacy, personalized medicine, hospital pharmacy, community pharmacy

## Abstract

The use of pharmacogenetics to optimize pharmacotherapy is growing rapidly. This study evaluates the feasibility and operability of a collaborative circuit involving hospital and community pharmacists to implement clopidogrel pharmacogenetics in Barcelona, Catalonia, Spain. We aimed to enroll patients with a clopidogrel prescription from cardiologists at the collaborating hospital. Community pharmacists collected patients’ pharmacotherapeutic profiles and saliva samples, which were then sent to the hospital for *CYP2C19* genotyping. Hospital pharmacists collated the obtained data with patients’ clinical records. Data were analyzed jointly with a cardiologist to assess the suitability of clopidogrel. The provincial pharmacists’ association coordinated the project and provided IT and logistic support. The study began in January 2020. However, it was suspended in March 2020 due to the COVID-19 pandemic. At that moment, 120 patients had been assessed, 16 of whom met the inclusion criteria and were enrolled in the study. The processing of samples obtained before the pandemic had an average delay of 13.8 ± 5.4 days. A total of 37.5% patients were intermediate metabolizers and 18.8% were ultrarapid metabolizers. No poor metabolizers were detected. Pharmacists rated their experience with a 7.3 ± 2.7 likelihood of recommending that fellow pharmacists participate. The net promoter score among participating pharmacists was +10%. Our results show that the circuit is feasible and operable for further initiatives.

## 1. Introduction

### 1.1. Pharmacogenetics and Pharmacy Practice

One third of patients do not adequately respond to pharmacological treatment or have potentially severe side effects [1]. Pharmacotherapeutic failure has a high economic impact on national health systems (NHS), and leads to a significant reduction in patients’ quality of life [2,3]. Genetic factors are determinant in drug response and explain much of the interindividual variability [4]. Consequently, there is currently great interest in conducting pharmacogenetic studies that contribute to the implementation of personalized medicine [5,6,7].

It is of the utmost importance that healthcare professionals (physicians, pharmacists, nurses, etc.) understand the role of genetic biomarkers in predicting drug response/toxicity due to their potential clinical relevance [8,9]. This cross-disciplinary knowledge will undoubtedly generate better health outcomes, greater quality of life for patients, and a more cost-effective use of drugs, which are essential to maintain national health care systems. Collaboration in this field is a first-order need to achieve as personalized a therapy as possible [8,9].

Pharmacogenetics has been incorporated into the daily care routine of several pharmacy departments in Spain, although they still represent but a minority [10,11]. As medication experts, hospital pharmacists are familiar with the factors that modulate drug pharmacokinetics and pharmacodynamics (age, sex, interactions with other drugs or herbal medicines, ethnicity, renal or liver function, etc.), and make recommendations based on these aspects. In addition, hospital pharmacists conduct pharmacokinetic studies of several drugs with a narrow therapeutic range and develop individualized dosing recommendations based on patients’ clinical characteristics [12]. Individualization based on genetic biomarkers, however, is much less known within this profession. For this reason, given the rise in genomic medicine, both the Spanish Society of Hospital Pharmacy (SEFH) and its Pharmacokinetics and Pharmacogenetics Working Group are currently promoting this area of knowledge [10].

Community pharmacists’ knowledge of pharmacogenetics is lower than that of their hospital pharmacy peers and, to date, their involvement in this field in Spain has been limited, despite the few pilot studies [13]. However, several projects have been implemented in countries such as the UK [14], the USA [15,16,17], Canada [18], and Switzerland [19] and are currently at various stages of development. A systematic review analyzing the implementation of pharmacogenomic testing in community pharmacies in the 2003–2021 period highlighted the barriers to pharmacogenetic implementation. Such barriers included pharmacists’ awareness of knowledge gaps, low confidence in interpreting and communicating pharmacogenetic results, and concerns about cost, privacy, and integration into the pharmacy workflow [20]. In Spain, several provincial pharmacist associations have developed training initiatives for their members to enable the application of pharmacogenetics into their daily practice whenever possible [21,22,23,24,25,26], and have funded projects to implement pharmacogenetics [27,28]. The project described here is the most recent initiative conducted in Spain aiming to integrate hospital and community pharmacists, as well as physicians.

### 1.2. Clopidogrel Pharmacogenetics

Clopidogrel is a commonly prescribed antiplatelet agent that acts as an irreversible selective inhibitor of ADP P2Y12 receptors in platelets [29,30]. This reduces the risk of cardiovascular and cerebrovascular ischemic atherothrombotic events in patients with atherosclerosis, and is slightly more effective than acetylsalicilic acid (ASA) [31,32,33]. The combination of clopidogrel and ASA reduces coronary risk in patients with non-ST-elevation acute coronary syndrome [34], patients with ST-elevation myocardial infarction [35], patients with atrial fibrillation who are not candidates for anticoagulation [36,37], and patients with acute minor stroke or transient ischemic attack [38].

*CYP2C19* is a key enzyme involved in clopidogrel’s liver bioactivation [39]. The recommended doses of clopidogrel are less effective in carriers of *CYP2C19* loss-of-function (LOF) variants. Therefore, genotyping is recommended in the clopidogrel datasheet to determine the most appropriate therapeutic strategy [29,30]. The most common LOF variant in the Spanish population is the *2 allele, with an allelic frequency of approximately 13% [40]. The *3 allele, another LOF variant, has a high frequency in patients from Asia and Oceania, but not in Spanish patients, where its allelic frequency is less than 0.1% [40]. Additionally, the presence of the CYP2C19*17 allele, which is associated with higher enzymatic activity, has been linked to an increased risk of bleeding, although the results reported to date are contradictory [29,30]. This allele is relatively common in Spain, with an allelic frequency of around 42% [40].

The Clinical Pharmacogenetics Implementation Consortium (CPIC) has made recommendations for antiplatelet therapy based on the *CYP2C19* genotype for patients with acute coronary syndrome undergoing percutaneous coronary interventions, such as stent placement [41]. Given the decreased effectiveness described for both intermediate metabolizers (genotypes *1/*2, *1/*3 and *2/*17) and poor metabolizers (*2/*2, *2/*3 and *3/*3) of *CYP2C19*, the CPIC recommends the use of an alternative antiplatelet agent (prasugrel or ticagrelor) in these cases, provided it is not contraindicated [41]. For ultrarapid metabolizers (genotypes *1/*17, *17/*17) or extensive metabolizers (genotype *1/*1), the CPIC recommends the standard dose of clopidogrel [41]. A patient is considered to have the *1/*1 genotype, with normal enzymatic activity, if they do not have any of the aforementioned alternative alleles (*2, *3 or *17). Despite CPIC recommendations, there is still debate about the usefulness of routinely implementing *CYP2C19* genotyping to guide clopidogrel prescription [42]. In fact, some meta-analyses have been unable to demonstrate the clinical utility of this genotyping [43,44]. Conversely, several recent studies show that prescribing clopidogrel according to *CYP2C19* genotyping results is cost-effective [45,46].

### 1.3. Aims and Justification

Based on the above and considering that clopidogrel is a commonly prescribed drug that is ordinarily dispensed in community pharmacies, we designed a collaboration circuit between hospital and community pharmacists to integrate the two settings in order to implement clopidogrel pharmacogenetics in clinical practice.

Our aim was to assess whether the implementation of pharmacogenetics in daily clinical practice was feasible and operational through cooperation and synergy between hospital pharmacies and community pharmacies. To consider overall efficiency, we evaluated the collaboration between the two healthcare settings, taking into account their advantages and risks. We hypothesized that collaboration between hospital and community pharmacists could lead to the establishment of an applicable and effective circuit for the implementation of pharmacogenetics. 

The main objectives of the study were:

1. To establish a pilot circuit in which pharmacogenetic markers (variants of *CYP2C19*) are determined in order to optimize the clopidogrel prescription based on the recommendations of international guidelines (CPIC).
2. To evaluate whether the established circuit is feasible and operational.

The secondary objectives of the study were:

1. To identify patients who are intermediate or poor metabolizers of *CYP2C19* who are currently taking clopidogrel, whose treatment will be individually evaluated.
2. To identify patients who are carriers of the *17 allele and evaluate whether they have an increased risk of bleeding.

## 2. Materials and Methods

### 2.1. Study Design

This prospective study established a novel circuit in which community and hospital pharmacists cooperated with geneticists and cardiologists to implement a pharmacogenetic test in routine care. The study, which was conducted in an urban area in Barcelona city (Catalonia, Spain), was led by the Hospital de la Santa Creu i Sant Pau (HSCSP) Pharmacy Department and the Barcelona Pharmacists’ Association (COFB is its abbreviation in Catalan). Participating community pharmacies were located in the reference area of this high-complexity tertiary hospital, which provides healthcare to 24% of the population of Barcelona (i.e., up to 407,550 inhabitants). Community pharmacies in the area were able to voluntarily choose to participate in the project when COFB invited them.

The COFB was responsible for the circuit logistics and the development of a cloud-based electronic case report form (eCRF) on Farmaserveis, the pharmaceutical care activity platform for community pharmacists in Catalonia [47]. Together with the HSCSP Genetics and Pharmacy Departments, they also organized specific training on pharmacogenetics for participating community pharmacists.

**Figure 1:**
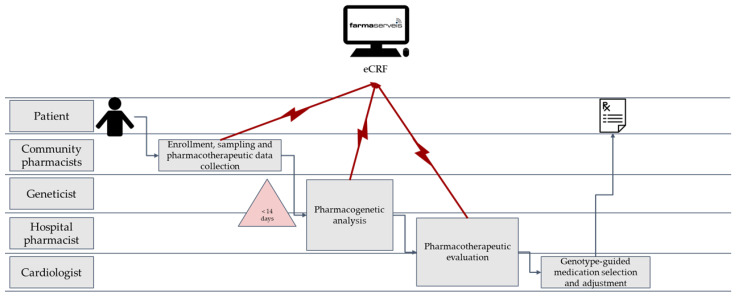
Value stream map of the circuit designed to implement clopidogrel pharmacogenetics in clinical practice.

Figure 1 shows a value-stream map [48] explaining the circuit and the implication of each participating stakeholder.

### 2.2. Healthcare Professionals’ Training for the Study

In November 2019, a meeting was held at COFB headquarters to present the project to interested community pharmacists. Those who decided to participate in the project underwent a five-hour training course on introductory concepts of pharmacogenetics, its clinical usefulness, the clinical role of the pharmacist in its development, and cytochrom P450 and clopidogrel pharmacogenetics. In the final stage of the training, each step of the process was discussed in detail and saliva extraction kits were provided to all participating pharmacists.

### 2.3. Study Population Sampling, Inclusion and Exclusion Criteria

The inclusion criteria were as follows: age ≥ 18 years old and a clopidogrel prescription made by an HSCSP cardiologist. The exclusion criteria were: a non-cardiac indication for clopidogrel, and hospitals other than HSCSP. When a patient fulfilled the inclusion criteria, the community pharmacist explained the study to them and provided written information so that they could provide their informed consent.

### 2.4. Ethical Considerations, Information for Subjects, and Informed Consent

The study was carried out strictly following international ethical recommendations for medical research involving human subjects. The researcher was responsible for ensuring that the study was carried out in accordance with the standards set out in the Helsinki Declaration. Before the study began, HSCSP Clinical Research Ethics Committee approved the study protocol (code: IIBSP-FAR-2019-29).

The objectives, methods, and potential risks of the study were explained to the potential participant or their legal guardian/family member in language that was comprehensible to lay persons. Before entering the study, consent was recorded with the signature of the subject or their legal guardian/family member.

### 2.5. Data Collection

After obtaining their consent, we electronically recorded the patients’ social and clinical data that were relevant to the study in the specific eCRF, located in Farmaserveis. This safe cloud-based web application coded by COFB IT Department [47] ensured the confidentiality of patients’ data and the security of its management, in accordance with current Data Protection legislation [49], and only professionals participating in the project had access to this.

The following variables were defined for the study: sociodemographic variables (age, sex, educational level, socioeconomic level, and ethnic group), and clinical variables (smoking habit, arterial hypertension, diabetes mellitus, dyslipidemia, family history of ischemic heart disease, personal history of ischemic heart disease, peripheral vascular disease, previous stroke, chronic kidney disease, *CYP2C19* genotype, indication for antiplatelet treatment, date of start and end of antiplatelet treatment, type of coronary artery disease, revascularization technique, left ventricular ejection fraction at discharge, thrombotic and hemorrhagic events during clopidogrel treatment, and collection of the patient’s pharmacotherapeutic history).

The indicators of the feasibility and operability of the circuit were: the percentage of participating community pharmacies that enrolled patients; the number of patients who refused to participate in the study; the average time from patient recruitment until the pharmacogenetic report; and the average time the sample took to reach the Research Institute.

At the end of the project, participating pharmacists gave their opinion regarding the circuit. We measured their opinion through the Net Promoter Score [50]. We calculated this score by subtracting the percentage of participating community pharmacists who scored 0–6 from the percentage of customers who scored 9 or 10 [50].

The information required for the development of the study was obtained as follows:

• An interview was held with the patient at the time of recruitment in the community pharmacy. As specified in the informed consent, in order to participate in the study, the patient had to provide the following data: NHS patient identification code, first name and surname, sex, date of birth, phone number, email address, zip code, nationality, and indication for the clopidogrel prescription and any other drugs taken.
• Medical history. All participants granted consent for hospital pharmacists and clinicians participating in the study to review their clinical history to extract data of interest regarding the abovementioned clinical variables.
• Online opinion survey. Participating pharmacists were sent a survey through a web-based system at the end of September 2020. The project was resumed briefly in November 2020 when the second peak in the COVID-19 wave had passed.

### 2.6. Sampling and Analytic Methods

A saliva sample was collected from all participating patients at the community pharmacy using the Danagene Saliva Kit (Danagen^®^, Barcelona, Spain). This saliva collection and preservation device provided a safe, simple, and fast procedure for collecting, stabilizing, and transporting saliva samples at room temperature. Samples were then sent to the Research Institute through a specialized carrier. DNA was then isolated and purified using the same kit. The concentration was measured using a NanoDrop instrument (Thermo Fischer, Waltham, MA, USA). The presence of alleles *2 and *17 was then determined using TaqMan hydrolysis probes (Thermo Fischer, Waltham, MA, USA) and real-time PCR technique (7500 Fast Real-Time PCR System instrument, Applied Biosystems, Waltham, MA, USA). The choice of these alleles was based on the frequency described in the Spanish population. Genotyping of other alleles (such as *3, *5 or *6) was discarded due to their low population frequency in Spain. The patient’s genotype was considered *1/*1 if neither allele *2 nor allele *17 were present.

All results were to be delivered within two weeks. They were then analyzed by geneticists and the study director, who prepared a report for each patient. The obtained genotype and its clinical meaning (ultrarapid, extensive (i.e., normal), intermediate, or poor metabolizer) was posted on the application. The community pharmacist was able to consult the results of patients recruited in their pharmacy and inform them of the study results. If the patient was not a *2 allele carrier, the community pharmacist informed the patient that, based on the genetic result, they could continue with the drug. If the patient was a carrier of at least one allele *2, the community pharmacist informed the patient that the cardiologist from the hospital center would assess the suitability of their treatment on an individual basis.

### 2.7. Data Management and Analysis

Project researchers, including community, hospital and COFB pharmacists, were responsible for creating the database, and entering and analyzing the data. For each patient, all the variables specified in the corresponding section were collected and anonymized for subsequent analyses. A descriptive analysis was carried out using frequencies, proportions, ranges, means, standard deviations, and 95% confidence intervals. Student’s *t*-test was performed, with statistical significance defined at 0.05%, when needed. The analyses were carried out with GraphPad Prism 5 for Windows, version 5.02 (GraphPad Sofware, Inc., launched in 2008, San Diego, CA, USA) and Microsoft Excel 2013 for Windows (Microsoft, Inc. launched in 2012, Redmond, WA, USA).

## 3. Results

Recruiting started on 13 January 2020. On 25 February 2020, the first case of COVID-19 was reported in Catalonia, and on 13 March 2020, a state of alarm, with lockdown and social distancing measures, was declared throughout Spain. As all healthcare resources were focused on the COVID-19 healthcare crisis, the project’s logistics were impaired. The results reported here pertain to the two months before lockdown and a month in November 2020, when the study was briefly resumed. The participating community pharmacists assessed 120 patients who had clopidogrel prescriptions. Of the 120 patients, 24 met the inclusion criteria (clopidogrel prescription by a HSCSP cardiologist) and 16 (67%) consented to enroll in the study. The 16 patients were recruited in 16 community pharmacies. Eight of the community pharmacies that participated in the study (*n* = 24) did not recruit any patient.

Table 1 shows the sex and age of the patients that were assessed and enrolled. Most participants were males and mean age was around 70 years old. All were Caucasian. Regarding education, 6.3% had no formal education. Primary school was the highest education level, seen in 50% of patients, 25% went to secondary school, 12.5% had vocational training and education, and 6.3% went to university.

Predictably, all patients had cardiovascular risk factors. In order of frequency, these were: dyslipemia, hypertension, tobacco smoking history, type-2 diabetes, familial and personal history of cardiac ischemia, stroke history, peripheral vascular disease and chronic kidney disease. The basal clinical characteristics of all enrolled patients are detailed in Table 2.

**Figure 2:**
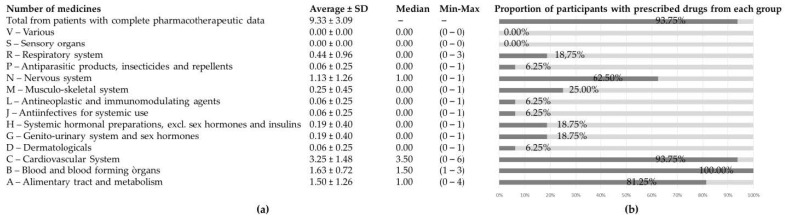
Pharmacotherapy of enrolled patients: (**a**) average number of medicines prescribed in total and within every ATC 1st–level group; and (**b**) proportion of participants who had at least one drug prescribed from every ATC 1st–level group.

Complete pharmacotherapeutic data were gathered for 93.8% of the participants. Clopidogrel was prescribed in all participants due to cardiopathy of an average of 4.7 ± 5.4 years (Median: 2.5). Notably, 50% of the participants had received clopidogrel for a year or less, 36% for between 1 and 8 years, and 14% for longer than 8 years (maximum of 17 years per participant). All patients were polymedicated, with an average of 9.3 ± 3.1 prescribed medicines per patient (4–15). Figure 2 shows information regarding the proportion of patients taking drugs from each ATC level, along with the quantity per patient. All participants had medicines from group B (Blood and blood-forming organs) corresponding to at least the clopidogrel prescription. The proportion of patients with prescriptions of drugs corresponding to group C (cardiovascular system) and group A (alimentary tract and metabolism) was also high. For groups A and C, the prescribed medications were mainly antidiabetic and antihypertensive drugs. Interestingly, up to 62.5% of participants had prescription drugs corresponding to group N (nervous system)—mainly benzodiazepines, analgesics and antiepileptic medications.

From the point of view of sample processing, logistics and analysis, a significant difference (*p* < 0.001) was found in the time that elapsed from sampling to analysis between samples collected before the first case of COVID-19 and those collected later: in the regular period before 25 February 2020, sample results took 13.8 ± 5.4 days. Thereafter, they took 84.4 ± 31.3 days. This difference was largely due to the increase in time taken from sampling to sample reception as a result of the reduction in mobility during lockdown in Spain (see Table 3). There were no differences regarding the quality of the samples, with an average concentration of 952 ± 542 ng/µL.

Genotyping results revealed that six patients (37.6%) were intermediate metabolizers, seven (43.8%) were normal metabolizers and the remaining three patients (18.8%) were ultrarapid metabolizers. No poor metabolizers (CYP2C19*2/*2 genotype) were found. Consequently, and following the CPIC guidelines, six patients, those who were intermediate metabolizers (*1/*2 or *2/*17 genotypes), were potential candidates to change clopidogrel treatment to another antiplatelet drug, such as prasugrel or ticagrelor (see Table 3). However, after review by their cardiologists, all six had contraindications, namely, a high risk of bleeding, for the prescription of a more potent drug, such as prasugrel or ticagrelor, and they continued on clopidogrel. Additionally, clopidogrel therapy was shown to be effective and had no adverse effects of note.

The opinion of 27% of participating community pharmacies was collected regardless of whether they had enrolled any patient. At least one patient was enrolled by 80% of respondent pharmacists. Table 4 shows the ratings given by the respondent pharmacists to the support received from the COFB Help Desk and the Research Institute to the eCRF and to the pharmacogenetics report generation system. To the question “How likely are you to recommend participation in this pharmacogenetic circuit to a fellow pharmacist? (0, not likely at all; 10, extremely likely)” the average rating was 7.3 ± 2.7. These data show that this circuit has a net promoter score [50] of +10%.

## 4. Discussion

To our knowledge, this is the first pharmacogenetic circuit in Spain involving hospital and community pharmacists and physicians, the key stakeholders in this multilevel and multidisciplinary project. A logistic and IT scheme was designed in this project to allow for stable collaboration among professionals providing pharmacogenetic services. This scheme enables the application of effective pharmacotherapeutic interventions according to patient genotype, as cardiologists can include pharmacogenetic information to guide their prescribing criteria. We found the experience to be satisfactory: no samples were lost, the isolated DNA quality was satisfactory, genotyping was successful for all patients and the average response time was adequate, at least in the pre-COVID-19 period.

Several aspects regarding the results of this study should be assessed. The results show that the implemented circuit is feasible, as it provides pharmacogenetic results in a reasonable period of time, allowing for a genotype-based intervention regarding patients’ prescriptions. Furthermore, community pharmacists considered their participation satisfactory in all the assessed elements (received support, eCRF application and pharmacogenetics report generation system), which is key to developing further similar initiatives. The lowest-rated element of the project was the pharmacogenetic report generation system, but this was approved with a rate of 5.8 out of 10. Since the suggestions reported by participating pharmacists have been taken into account, several elements of this report generation system have been improved to meet their needs.

Our results show that no clopidogrel prescriptions were changed after *CYP2C19* genotyping. This could be due to several factors: first, the number of patients recruited was relatively low due to the COVID-19 pandemic. It is likely that some clopidogrel prescriptions would have been changed in a larger sample. Second, according to the guidelines in our region, only patients who had a myocardial infarction and present contraindications to prasugrel or ticagrelor (because of their advanced age, the existence of previous hemorrhagic episodes, etc.) are prescribed clopidogrel. This suggests that a few identified carriers of the *2 allele may be candidates for an alternative treatment. Interestingly, and in keeping with our results, in the study by Ferreri et al. in the USA, no clopidogrel prescriptions were changed to prasugrel or ticagrelor [51] despite the CPIC recommendations for intermediate/poor *CYP2C19* metabolizers [41].

From our point of view, the pharmacists involved in pharmacogenetics could guarantee the usefulness and reliability of these genetic tests and their correct interpretation, as was pointed out in a recent review [52]. Of note, the ASHP Statement on the Pharmacist’s Role in Clinical Pharmacogenomics, published in 2022, stated that the data-interoperability and data-sharing of pharmacogenomic test results with other healthcare institutions, including community pharmacies, would be a critical factor to enable the continued use of the information over a patient’s lifetime [53]. On these grounds, we consider that the initiative reported here is a valuable contribution to this objective.

Pharmacists’ involvement in pharmacogenetics may be complimentary to several more traditionally pharmacist-led interventions, such as medication acquisition, adverse reaction monitoring and patient education [52]. Some authors also highlight the relevance of involving community pharmacists in pharmacogenetics services, but they emphasize the need for appropriate funding or reimbursement to ensure the economic sustainability of these services [54]. The provision of the pharmacogenetic services in the present circuit was funded by research grants and a sponsor (see the Funding section below). However, we believe that these pharmacogenetics services should be funded by the NHS to ensure their continuity. Certainly, such funding would only be offered for well-defined patient profiles that could benefit from such an approach in a cost-effective manner [45,46]. Currently, pharmacogenetics in Spain is funded by the NHS, but is primarily limited to certain treatments (particularly oncological) whose dispension is restricted to hospital pharmacists [10]. In our opinion, this funding should also be considered for drugs dispensed in community pharmacies in which pharmacogenetics plays a role.

As mentioned earlier, this is the first project in Spain in which hospital pharmacists, community pharmacists and prescribers collaborated to implement pharmacogenetics in clinical practice. The only previous Spanish community pharmacy-based experience was a pilot study carried out in 2013. This included two interventions: a pharmacotherapeutic review with follow-up by community pharmacists and pharmacogenetic analyses of *CYP2C9* and *CYP3A4* in antihypertensive therapy. It was performed in a hospital setting, similarly to our study [13]. As the pharmacogenetic tests were not performed until all patients were recruited, genotype-based interventions were not an option [13]. In the USA, Ferreri et al. conducted a similar study to ours in 2014 [51], in which 18 patients taking clopidogrel were recruited in a community pharmacy for *CYP2C19* genotyping. However, the circuit implemented in the US American healthcare system (based on private insurance and personal out-of-pocket funding) is not fully comparable to our healthcare system, which is based on an integrated and decentralized, single-payer Beveridge model with universal coverage [55]. In a more recent study, in 2023, seven European countries, including Spain, participated in an open-label, multicenter, controlled, cluster-randomized crossover study to implement a preemptive 12-gene pharmacogenetic panel. This study involved 18 hospitals, 9 community health centers, and 28 community pharmacies. However, no Spanish community pharmacies participated [56]. It is noteworthy that point-of-care pharmacogenetic testing may acquire great importance in the future. Such platforms can deliver results within a short time, without requiring follow-up appointments [57]. This technology has the potential to simplify the logistics of our circuit. Nevertheless, the cost of infrastructure needs to decrease to enable its widespread adoption [57].

Our study has several limitations. First of all, like many other projects that took place at the time, the circuit was heavily impacted by the COVID-19 pandemic, especially from the logistic perspective. The Spanish government-declared state of alarm started on 13 March 2020 [58], and included a generalized lockdown with a curfew that lasted for 100 days. This lockdown made the total time from sampling to analysis increase from less than two weeks to more than 80 days. Fortunately, DNA samples were sufficiently stable to remain adequate for analysis after such a long period of time. However, taking into account the pre-COVID-19 time spent on sample processing, logistics and analysis show this is completely feasible and that the circuit was operational in a timely manner. It was also operational with a stressor such as the COVID-19 healthcare crisis. Second, the sample size proposed for collection is relatively small. It should be noted that it was difficult to calculate how many patients with a clopidogrel prescription initially made by a HSCSP cardiologist would go to a pharmacy in their reference area that also voluntarily decided to participate in the project using the available preanalytical data. Third, according to the clinical guidelines in our region, clopidogrel is generally prescribed for patients who have a myocardial infarction and contraindications to prasugrel or ticagrelor, such as advanced age or previous hemorrhagic episodes. Consequently, most intermediate or poor *CYP2C19* metabolizers have contraindications to prasugrel or ticagrelor. Our results refer to the three-month period (January, February and November) of implementation in 2020.

## 5. Conclusions

In conclusion, our project shows that implementing a circuit for a pharmacogenetics service in clinical practice is feasible and operational, with community pharmacists working together with hospital pharmacists, geneticists and clinicians. This collaborative initiative enhanced several operational features of the circuit, increasing not only its usefulness but also the overall satisfaction of the patients and healthcare professionals involved.

## Figures and Tables

**Table 1:** Age and sex in enrolled and assessed patients.

	Enrolled Patients			Assessed Patients		
**Total patients**	N = 16			N = 120		
**Gender**	***n***	**%**		***n***	**%**	
Male	11	68.8%		71	59.2%	
Female	5	31.2%		49	40.8%	
	**Avg ± SD**	**Median**	**Range** **(Min–Max)**	**Avg ± SD**	**Median**	**Range** **(Min–Max)**
**Age**	71.8 ± 12.4	73.0	(51–94)	68.3 ± 13.3	72.0	(41–95)

**Table 2:** Basal clinical characteristics of the enrolled patients.

Total Enrolled Patients	N = 16	
**Tobacco use**	***n***	**%**
Smoker	1	6.3%
Former smoker	8	50.0%
Never smoker	7	43.8%
**Cardiovascular risk factors**	***n***	**%**
Hypertension	10	62.5%
Dyslipemia	16	100.0%
Type-2 diabetes	8	50.0%
Family history of cardiac ischemia	7	43.8%
Personal history of cardiac ischemia	6	37.5%
Personal history of stroke	2	12.5%
Peripheral vascular disease	2	12.5%
Chronic kidney disease	1	6.3%
**Clinical situation**	***n***	**%**
ST-elevation myocardial infarction	3	18.8%
Non-ST-elevation myocardial infarction	1	6.3%
Chronic angina	3	18.8%
Unstable angina	3	18.8%
Unavailable data	6	37.5%
**Coronary disease type**	***n***	**%**
1 blood vessel	3	18.8%
2 blood vessels	2	12.5%
Truncus arteriousus	1	6.3%
Unavailable data	10	62.5%
**Coronary revascularization technique**	***n***	**%**
Drug-eluting stents	5	31.3%
Conventional stents	2	12.5%
Surgical intervention	3	18.8%
None	4	25.0%
Unavailable data	2	12.5%
**Other clinical information**	**Avg ± SD**	**Median**
Left ventricle ejection fraction	39.7 ± 30.5	42.0
Number of thrombotic events	0.00 ± 0.00	0.00
Number of hemorragic events	0.29 ± 0.47	0.00

**Table 3:** Sample processing, logistics and analysis.

Time from Sampling to Lab Sample Reception (Days)	Avg ± SD	Median	Range (Min–Max)	
Pre-COVID-19 period	7.7 ± 5.5	7.0	(1–18)	*p* < 0.001
Post-COVID-19 period	64.1 ± 36.8	85.0	(7–91)	
**Time from Reception of Lab Sample to Analysis (days)**	**Avg ± SD**	**Median**	**Range** **(Min–Max)**	
Pre-COVID-19 period	6.1 ± 1.1	7.0	(5–7)	*p* = 0.099
Post-COVID-19 period	20.3 ± 31.9	11.0	(0–92)	
**Total Time from Sampling to Analysis (days)**	**Avg ± SD**	**Median**	**Range** **(Min–Max)**	
Pre-COVID-19 period	13.8 ± 5.4	14.0	(8–23)	*p* < 0.001
Post-COVID-19 period	84.4 ± 31.3	96.0	(14–102)	
***CYP2C19* Genotype (Expected Phenotype)**	***n***	**%**		
CYP2C19*1/*1 (normal metabolizer)	7	43.75%		
CYP2C19*1/*17 (ultrarapid metabolizer)	3	18.75%		
CYP2C19*1/*2 (intermediate metabolizer)	5	31.25%		
CYP2C19*2/*17 (intermediate metabolizer)	1	6.25%		

**Table 4:** Opinion of participating community pharmacists regarding the circuit.

How Do You Rate... (from 0 to 10)	Avg ± SD	Median	Range (Min–Max)
... the support received from the COFB Help Desk?	8.7 ± 0.9	9.0	(7–10)
... the support received from the Research Institute?	7.1 ± 2.5	8.0	(2–9)
... the electronic case report form in Farmaserveis App?	6.8 ± 2.1	7.5	(3–9)
... the pharmacogenetic report generation system?	5.8 ± 2.6	6.5	(2–9)
**How likely is it that you would recommend a fellow pharmacist to participate in this pharmacogenetic circuit?**			
(0, not likely at all; 10, extremely likely)	7.3 ± 2.7	7.5	(2–10)
**Distribution of respondent groups**	***n***	**%**	
Promoter pharmacists (10–9)	4	40%	
Passive pharmacists (8–7)	3	30%	
Detractor pharmacists (6–0)	3	30%	
**Net Promoter Score (from −100% to +100%)**	**+10%**		

## Data Availability

The data presented in this study are available on request from the corresponding author. The data are not publicly available to ensure the confidentiality of patient’s data and the security of its management, in accordance with the current Spanish Data Protection legislation.
